# Aberrant Promoter Hypermethylation of RASSF Family Members in Merkel Cell Carcinoma

**DOI:** 10.3390/cancers5041566

**Published:** 2013-11-18

**Authors:** Antje M. Richter, Tanja Haag, Sara Walesch, Peter Herrmann-Trost, Wolfgang C. Marsch, Heinz Kutzner, Peter Helmbold, Reinhard H. Dammann

**Affiliations:** 1 Institute for Genetics, University of Giessen, Giessen D-35392, Germany; E-Mails: Antje.Richter@gen.bio.uni-giessen.de (A.M.R.); Tanja.Haag@gen.bio.uni-giessen.de (T.H.); sara.walesch@gen.bio.uni-giessen.de (S.W.); 2 Institute of Pathology, Halle D-06097, Germany; E-Mail: pherrmanntrost@pathologie-halle.de; 3 Department of Dermatology, University of Halle, Halle D-06120, Germany; E-Mail: wolfgang.marsch@medizin.uni-halle.de; 4 DermPath, Friedrichshafen D-88048, Germany; E-Mail: kutzner@w-4.de; 5 Department of Dermatology, University of Heidelberg, Heidelberg D-69120, Germany; E-Mail: Peter.Helmbold@med.uni-heidelberg.de

**Keywords:** merkel cell, tumor suppressor, DNA methylation, epigenetics, RASSF

## Abstract

Merkel cell carcinoma (MCC) is one of the most aggressive cancers of the skin. RASSFs are a family of tumor suppressors that are frequently inactivated by promoter hypermethylation in various cancers. We studied CpG island promoter hypermethylation in MCC of RASSF2, RASSF5A, RASSF5C and RASSF10 by combined bisulfite restriction analysis (COBRA) in MCC samples and control tissue. We found RASSF2 to be methylated in three out of 43 (7%), RASSF5A in 17 out of 39 (44%, but also 43% in normal tissue), RASSF5C in two out of 26 (8%) and RASSF10 in 19 out of 84 (23%) of the cancer samples. No correlation between the methylation status of the analyzed RASSFs or between RASSF methylation and MCC characteristics (primary *versus* metastatic, Merkel cell polyoma virus infection, age, sex) was found. Our results show that RASSF2, RASSF5C and RASSF10 are aberrantly hypermethylated in MCC to a varying degree and this might contribute to Merkel cell carcinogenesis.

## 1. Introduction

Merkel Cell Carcinoma (MCC) is a rare but aggressive cutaneous malignancy [1] of the elderly with poor prognosis [2]. The malignancy is believed to originate from neuroendocrine cells of the skin. These are found in the dermis of the skin as nerve-associated neuroendocrine cells and in the basal layer of the epidermis of the skin (Merkel cells) [3]. Little is known regarding the molecular mechanism underlying MCC development. It was reported that Merkel Cell Polyomavirus (MCPyV) shows presence in MCCs [4,5,6,7] and viral DNA was integrated within the tumor genome in a clonal pattern [5,8]. It was suggested that MCPyV infection is acquired through close contact, possibly involving saliva and/or the skin [9]. Interestingly MCPyV presence was not only found in skin, but also in lung cancer. We have shown RASSF1A promoter methylation and MCPyV presence in small cell lung cancer [10]. In non-small cell lung cancer MCPyV presence was found together with increased BRAF and decreased Bcl2 levels [11]. In MCC however no BRAF(V600E) mutations were found and it was suggested that the classical MAP kinase signal transduction pathway is inactive [12], but mutation of the tumor suppressor p53 in MCC was reported [13]. Additionally we and others have shown promoter hypermethylation of the tumor suppressors p14ARF in 42% [14] and RASSF1A in 51% [7] of MCCs. Future studies are needed to elucidate underlying mechanism(s) that drive MCC development and progression.

Tumor suppressor genes (TSG) are commonly inactivated by promoter hypermethylation in cancer. Methylation occurs on the DNA level at 5' position of cytosines, when found as dinucleotides with guanine. CpGs are overrepresented in the promoter region of TSG forming so called CpG islands. DNA methylation in CpG islands of TSG leads to epigenetic silencing of the according transcript (as reviewed in [15,16]). Well studied epigenetically inactivated TSG are the RASSFs. RASSF abbreviates Ras-association domain family and all ten members are characterized by a Ras-association domain either *C*-terminally (RASSF1–6) or *N*-terminally (RASSF7–10). The functions of the *C*-terminal or classical members range from apoptosis induction, cell cycle inhibition to microtubule stabilization [17]. 

The focus of our current work was on RASSF2, RASSF5A, RASSF5C and RASSF10, due to the fact that epigenetic inactivation of these tumor suppressors of the RASSF family was already reported in different cancer types. We and others showed promoter hypermethylation of RASSF2 [18,19,20], RASSF5A [21], RASSF5C [22] and RASSF10 [23,24,25,26,27,28]. For the most prominent family member RASSF1A we already reported strong promoter hypermethylation in MCC [7], which was in accordance with its status of a very well characterized epigenetically inactivated tumor suppressor [17]. 

Our aim in the present study was the comparative analysis of RASSF promoter hypermethylation in a set of primary Merkel cell carcinoma and controls. Therefore the promoter regions of RASSF2, RASSF5A, RASSF5C and RASSF10 were analyzed by combined bisulfite restriction analysis (COBRA). We show that the degree of promoter hypermethylation in MCC varies between the RASSFs, but is present for different RASSFs at the same time.

## 2. Experimental

### 2.1. CpG Island Prediction, PCR Product Size and Digestion Products

The promoter regions of RASSF2, RASSF5A, RASSF5C and RASSF10 were analyzed by CpG plot [29] to show the existence of a CpG island. Primers for bisulfite treated DNA were designed to bind only fully converted DNA and amplify promoter region of specific RASSFs (listed in Table S1). Promoter region was chosen for CpG content and presence of according restriction enzymes. COBRA PCR product for RASSF2 is 167 bp with *Taq*1 sites at position 111. For RASSF5A COBRA PCR product is 334 bp after semi-nested PCR with *Taq*1 sites at position 94, 234 and 260. COBRA PCR product of RASSF5C is 322 bp after nested PCR with *Taq*1 sites at position 221 and 284. The RASSF10 COBRA PCR product is 241 bp (with *Taq*1 sites at 50 and 141) or with alternative primer pair 167 bp (with *Taq*1 sites at 67). A Summary of COBRA PCR products, CpG islands, primer positions and *Taq*1 restriction sites is shown in Figure S1.

### 2.2. Merkel Cell Carcinoma and Controls

We used retrospectively sampled Merkel cell carcinomas from the tumor registries of the University of Heidelberg, the University of Halle (Saale), DermPath Friedrichshafen, and the Institute of Pathology of Halle (Saale). 87 samples of 85 tumors (53 primary MCC, 12 local skin recurrent MCC, 22 MCC skin tumors with uncertain primary/recurrence decision) from 79 patients (76.2 ± 10.8 years; male/female ratio 0.75) were studied (Table S2). Skin control samples (n = 20) were obtained from the skin of surgical surplus areas of routinely-excised and histologically controlled benign nevus cell nevi or benign cysts. A minimum distance of 5 mm to the lesions as well as exclusion of histological detectable contaminations by cells of the excised lesions or inflammatory infiltrate was guaranteed. MCC were diagnosed histologically by a set of neuroendocrine markers including cytokeratin 20 (CK20) and thyroid transcription factor (TTF)-1 expression. We used corresponding sections of the paraffin-embedded material for hematoxylin and eosin staining, immunostaining and DNA isolation. Merkel cell polyoma virus (MCPyV) infection was investigated in all tumor and control samples as stated previously [7]. Eighty out of 87 (92%) of the MCC samples and seven out of 20 (35%) of the control samples showed MCPyV expression (Table S2).

### 2.3. DNA Isolation

Tissue specimens were deparaffinized by xylene and ethanol treatment. DNA was isolated with a QIAamp DNA extraction kit (Qiagen, Hilden, Germany) after a proteinase K restriction and concentrations of DNA were determined by UV-photospectrometery.

### 2.4. Methylation Analysis by COBRA

Genomic DNA from MCC or control tissue (2 µg) was bisulfite treated (12 µL 0.1 M hydroquinone, 208 µL 1.9 M sodium metabisulfite and pH 5.5 with NaOH) and incubated overnight at 50 °C. Then DNA was purified using MSB Spin PCRapace (STRATEC Molecular, Berlin, Germany), eluted in 50 µL H_2_O and followed by 10 min incubation with 5 µL 3 M NaOH at 37 °C. DNA was then precipitated with 100% ethanol and 7.5 M ammonium acetate and redissolved in 1 × TE buffer. 200 ng were subsequently used for 25 µL PCR reaction with COBRA primers (listed in Table S1). The PCR product was digested with 0.5 µL of *Taq*1 (Fermentas GmbH, St. Leon-Rot, Germany) 1 h at 65 °C and resolved on 2% TBE gel together with mock digest.

## 3. Results

The RASSF family members RASSF2, RASSF5A, RASSF5C and RASSF10 all contain CpG islands in their promoter region, as analyzed by CpG plot and UCSC Genome browser and as shown in Figure S1. The CpG islands lengths are approximately 1.1 kb for RASSF2, 1.2 kb for RASSF5A, 0.5 kb for RASSF5C and 2.3 kb for RASSF10. COBRA methylation analysis primers were placed within this region. We used COBRA technique for methylation analysis of the indicated promoters in MCCs and control tissues (Figures S1). 

**Figure 1:**
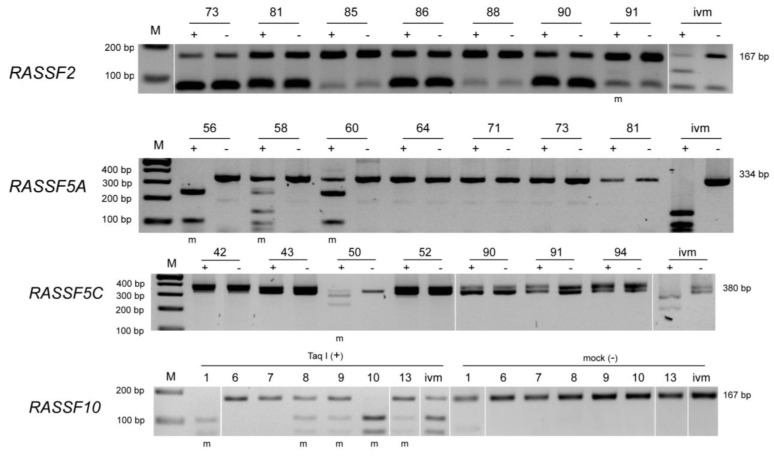
COBRA methylation analyses of RASSF2, RASSF5A, RASSF5C and RASSF10 in tumor tissue. Methylation analysis by COBRA for RASSF2, RASSF5A, RASSF5C and RASSF10 was performed and representative results are shown for different tumor samples (numbers are indicated above each gel). DNA was bisulfite treated and COBRA PCR with according primers was performed. Mock (−) and *TaqI* (+) digested PCR products are resolved in 2% TBE agarose gel together with 100 bp marker. An *in vitro* methylated (ivm) DNA was used as positive control. Methylated samples (m) are indicated below and PCR product sizes are shown beside picture.

**Figure 2:**
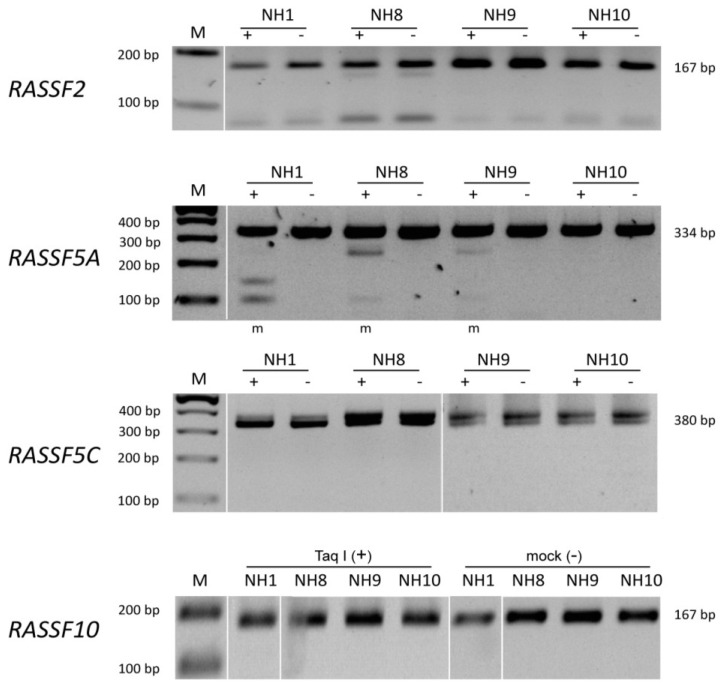
COBRA methylation analyses for RASSF2, RASSF5A, RASSF5C and RASSF10 in control tissue. Methylation analysis by COBRA for RASSF2, RASSF5A, RASSF5C and RASSF10 was performed and representative results are shown for different normal tissues (numbers are indicated above each gel). DNA was bisulfite treated and COBRA PCR with according primers was performed. Mock (−) and *TaqI* (+) digested PCR products are resolved in 2% TBE agarose gel together with 100 bp marker. Methylated samples (m) are indicated below and PCR product sizes are shown beside picture.

CpG island promoter methylation was analyzed for RASSF2, RASSF5A, RASSF5C and RASSF10 in Merkel cell tumor (Figure 1) and skin control tissue (Figure 2). 

DNA samples were bisulfite treated and COBRA PCR was performed. The PCR product was digested by restriction enzyme and resolved in TBE gel. An *in vitro* methylated DNA (ivm) was used as positive control. Representative samples are shown for each RASSF promoter analyzed. In case of RASSF2 promoter methylation in MCCs no sample shows digestion products as compared to ivm DNA (Figure 1). In control tissue all samples for RASSF2 are unmethylated and therefore undigested (Figure 2). RASSF5A is methylated in samples 56, 58, and 60 (Figure 1), however also control tissue shows some degree of methylation in samples NH1, NH8 and NH9 (Figure 2). RASSF5C is methylated in sample 50 (Figure 1), but not in any of the control samples (Figure 2). Interestingly, RASSF10 promoter analysis shows methylation in samples 1, 8, 9, 10 and 13 (Figure 1), but remains completely unmethylated in control tissue (Figure 2).

Table 1 summarizes the degree of promoter hypermethylation for the analyzed RASSF members in Merkel cell carcinoma tumor samples *versus* control tissue. For the RASSF2 promoter a total of 43 tumor samples were analyzed and 7% of these showed hypermethylation. The nine control tissue samples were all unmethylated. The RASSF5A promoter was studied in 39 tumor samples, of which 17 were methylated (44%). The according control tissue also showed a high degree of RASSF5A promoter methylation (43%). RASSF5C is methylated to a degree of 8% in 26 of tumor samples and unmethylated in control tissue. The RASSF10 CpG island promoter methylation was studied in 84 Merkel cell tumor samples of which 19 were methylated. Control tissue (14 samples) was unmethylated at the RASSF10 promoter region. 

**Table 1:** Summary of methylation analysis.

Gene	Tumor samples	Control samples
*RASSF2*	7% (3/43)	0% (0/9)
*RASSF5A*	44% (17/39)	43% (3/7)
*RASSF5C*	8% (2/26)	0% (0/11)
*RASSF10*	23% (19/84)	0% (0/14)

For details of tumors analyzed regarding MCPyV status and promoter methylation results see Table S2. We found neither a intercorrelation between the promoter methylation statuses nor between the methylation states of each of the investigated promoters and tumor type (primary/metastatic), MCPyV expression, age or sex, respectively (Pearson correlation, *p* > 0.05). 

## 4. Discussion

The aim of this work was to perform a comparative analysis of CpG island promoter hypermethylation of four Ras-association domain family members in Merkel cell carcinoma. MCC represents one of the most aggressive kinds of skin cancer [1], of which the underlying molecular mechanisms are poorly understood. The RASSF family consists of 10 members [17]. The most prominent family member is RASSF1A, which we already showed to be hypermethylated in MCC [7]. Therefore we studied further RASSF members regarding their promoter methylation state in MCC to test a possible common contribution to Merkel cell cancer formation. The members RASSF2, RASSF5A, RASSF5C and RASSF10 were chosen for this study. The remaining family members were excluded, either because members were previously reported to be only rarely or never epigenetically inactivated in cancer or did not contain a CpG island promoter at all [17,30]. 

The RASSF members analyzed in this study have in common a frequent promoter hypermethylation of their CpG island in cancer. The RASSF2 transcript was detected in normal tissue [31], but was shown to be down regulated by hypermethylation of its promoter region in various tumor entities [18,19,20,31,32,33,34]. RASSF5 exists in different isoforms as a result of alternative spicing and differential promoter usage. Isoforms A and C are transcribed from two separate promoters [35]. Both are expressed in normal tissue, but down-regulated in cancer cell lines [35,36,37,38]. We and others found promoter methylation of RASSF5A [19,21] and RASSF5C [22] in tumors. RASSF10 was shown to be hypermethylated at its CpG island promoter region in various tumor types [23,24,25,26,27,28] for instance malignant melanoma [25]. 

The RASSF members chosen for analysis were shown to harbor different functional properties. The mouse model suggests a role of RASSF2 in bone development [39], but also tumor suppressive properties were reported [20,30]. It was demonstrated that RASSF5A has growth suppressive activities [40] and RASSF5C was shown to play a role in lymphoid organs in the mouse system [41]. RASSF10 functions as a tumor suppressor [24,26,42] and additionally we demonstrated a possible role in cell differentiation [24]. 

In our study we showed that the RASSF2 promoter region was hypermethylated in 7% of cancer samples, but unmethylated in control tissue. With a total of 43 analyzed tumor samples RASSF2 seems to be hypermethylated in only a small subset of samples. RASSF5A was found to be frequently hypermethylated in Merkel cell carcinoma (44%), but also in control tissue (43%). Therefore promoter hypermethylation of RASSF5A seems not to be restricted to MCC. However, previous studies showed that RASSF5A can be epigenetically inactivated by promoter hypermethylation in other cancer types [21]. The RASSF5C promoter showed hypermethylation to a degree of 8% in Merkel cell carcinoma, but remained unmethylated in control tissue. Similar to RASSF2, only a small number of tumor samples harbor a RASSF5C promoter hypermethylation. The contribution of RASSF2 and RASSF5C promoter hypermethylation to MCC development and progression remains to be addressed. Most notable is the RASSF10 promoter methylation status. We detected 19 out of 84 primary MCC samples to be hypermethylated at the RASSF10 promoter in comparison to none out of 14 investigated control samples. We earlier reported that RASSF1A methylation was a frequent event and reached 51% in MCC [7], and in this study we show that also the RASSF10 promoter is frequently hypermethylated in MCC (23%). It is interesting that two members of the tumor suppressor RASSF family are hypermethylated in MCC. Though no correlation between methylation of these two RASSF was found in MCC, possibly due to the limited number of samples, it will be interesting to clarify if hypermethylation of both promoters drives MCC progression. It could be suggested that RASSF1A as a member of the *C*-terminal and RASSF10 as a member of the *N*-terminal family contribute to carcinogenesis through independent mechanisms. 

To date only limited functional data exist for RASSF10. However it was shown that the RASSF10 promoter was hypermethylated in cancers. e.g., malignant melanoma of the skin [25]. The current study adds Merkel cell carcinoma to the cancer types showing RASSF10 promoter methylation. Future studies should concentrate to confirm RASSF10 down regulation by promoter hypermethylation in MCC, as epigenetic inactivation of RASSF10 was earlier reported in different cancer cell lines and primary tumors [24,26,27,42,43].

## 5. Conclusions

The present study is the first comparative analysis of RASSF promoter methylation in Merkel cell carcinoma. In summary we were able to show different RASSFs like RASSF2, RASSF5C and RASSF10 are tumor specifically methylated at their promoter region in MCC, additional to the earlier reported presence of RASSF1A hypermethylation in MCC [7]. Promoter hypermethylation of tumor suppressors in cancer is a common and early mechanism of their inactivation, which can contributes to cancer progression [44]. Therefore detection of epigenetic changes like DNA methylation markers might prove useful in the early detection of cancer. It will be interesting to extend analysis to a larger set of MCCs to elucidate if correlations between RASSF members and/or clinical parameters are present. Additionally it will be fascinating to study the contribution of several inactive tumor suppressive RASSFs to Merkel cell carcinogenesis.

## Abbreviations

MCC: Merkel cell carcinoma
RASSF: Ras-Association Domain Family
TSG: tumor suppressor gene
COBRA: combined bisulfite restriction analysis

## Supplementary Files

Supplementary File 1 Supplementary Materials (PDF, 498 KB)
