# Supplementary material for: Aberrant Promoter Hypermethylation of RASSF Family Members in Merkel Cell Carcinoma
Source: Cancers (Basel). 2013 Nov 18;5(4):1566–76. doi: 10.3390/cancers5041566 (PMC3875954; doi:10.3390/cancers5041566)
Supplement: Supplementary File 1 — Supplementary Materials (PDF, 498 KB) [file cancers-05-01566-s001.pdf]

Supplementary Materials

Figure S1. Promoter structure of RASSF2, RASSF5A, RASSF5C and RASSF10 for methylation analysis. RASSF family members RASSF2, RASSF5A, RASSF5C and RASSF10 are shown with their CpG island promoter structure. Black vertical lines represent single CpGs and restriction enzyme *TaqI* recognition sites are marked with star. Bent arrows indicate transcriptional start sites. Horizontal arrows mark PCR product and size for COBRA methylation analysis. The according PCR fragment size is indicated.

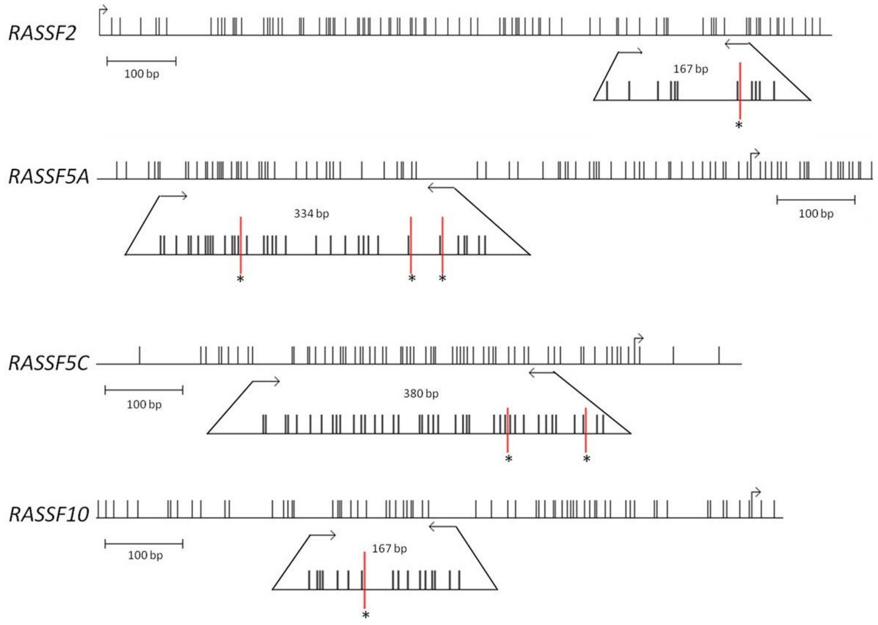

Table S1. Primer and PCR conditions.

Primer			5'–3'	PCR product size in bp	Restriction fragments
RASSF2	Upper		GATGGGAAGGYGTTTTTATTTTATTTT	167	111 + 56
	Lower		AAAACTAAAACCTACCTCTAAAAAATCC		
RASSF5A	Upper	semi-nested	GGATAGTTTTGTTAGTTTTTGGAGGTATT	(363) 334	94 + 140 + 26 + 74
	Lower		ACCCTAAACCTTCAACCCTACCTCTT		
	Lower		CTTACCAATCACTTTCCCCAACAC		
RASSF5C	Upper	nested	GGTTTTGAGGAATTTTGTAGAGGAA	(380) 322	221 + 63 + 38
	Upper		AGGAAGTGGTTTTAGAATTGTTTTA		
	Lower		AAAAAAAATAAACACCCCTCCCC		
	Lower		TAAACCCCTAACTCTAAACCCC		
RASSF10	Upper		ATAAGTAGAGGAGTTAGTAGGTTAAAGGAGA	241	50 + 91 + 100
	Upper		GTGGAGGGATTTTGAATTTTTTTT	167	67 + 100
	Lower		AAATACAAAAAACTCAAAACCCAAACCC		

Table S2. Tissue number, type of MCC (primary or recurrence), MCPyV and methylation status.

No	Patient	Tumor	Sample	Origin	MCPyV	RASSF2	RASSF5A	RASSF5C	RASSF10
MCC 1	1			P	+	-	-	-	+
MCC 3	2			P	+	-	-	-	+
MCC 6	3			P	+	-	-	-	0
MCC 7	4			R	0	-	-	-	0
MCC 8	5			P	+	-	-	-	+
MCC 9	6			P	+	-	-	-	+
MCC 10	7			P	+	-	-	-	+
MCC 11	8			P	+	-	-	-	0
MCC 13	9			P	+	-	-	-	+
MCC 14	10	1		R	+	-	-	-	+
MCC 15		2		R	+	-	-	0	0
MCC 16	11			P	0	-	-	-	+
MCC 17	12			P	+	-	-	-	0
MCC 18	13			P	+	-	-	-	+
MCC 20	14			P	+	0	-	0	0
MCC 21	15			P	+	0	0	0	0
MCC 22	16			P	+	-	+	0	0
MCC 23	17			P	+	0	0	-	0
MCC 24	18			P	+	-	-	-	0
MCC 25	19			R	+	-	-	-	0
MCC 26	20			P	0	-	-	-	+
MCC 28	21			P	+	-	-	-	0
MCC 29	22			R	+	-	-	-	0
MCC 30	23			P	+	0	0	0	+
MCC 31	24			P	+	0	+	+	+
MCC 33	25			P	+	-	-	-	0
MCC 35	26			P	+	0	0	0	0
MCC 36	27			P	+	0	0	0	0
MCC 37	28			R	+	0	-	-	0
MCC 38	29			P	+	0	0	0	0
MCC 39	30			P	+	0	0	0	+
MCC 40	31			R	+	-	-	-	0
MCC 41	32			P	+	-	-	-	0
MCC 42	33			P	+	0	+	0	0
MCC 43	34			P	+	0	+	0	0
MCC 44	35			R	+	-	-	-	0
MCC 45	36			P	+	0	-	-	-
MCC 46	37	1		P	+	-	0	-	0
MCC 47		2		R	+	-	0	-	+
MCC 48	38			P	+	-	-	-	0
MCC 49	39			P	+	+	+	-	+
MCC 50	40			P	+	0	0	+	0
MCC 51	41			R	+	0	+	-	0
MCC 52	42			R	+	-	+	0	0

Table S2. *Cont.*

No		Patient	Tumor	Sample	Origin	MCPyV	RASSF2	RASSF5A	RASSF5C	RASSF10
MCC	54	43			U	+	0	+	-	0
MCC	55	44	1		U	+	-	-	-	0
MCC	56		2		U	+	-	+	-	0
MCC	57	45			U	+	-	-	-	+
MCC	58	46			U	+	-	+	-	0
MCC	59	47	1		U	+	-	-	-	0
MCC	60		2		U	+	-	+	-	+
MCC	61	48			U	+	0	-	-	0
MCC	62	49			U	+	-	-	-	0
MCC	64	50			U	+	-	0	-	0
MCC	65	51			U	+	-	-	-	0
MCC	67	52			U	+	0	-	-	0
MCC	68	53			U	+	0	-	-	0
MCC	69	54			U	+	-	-	-	0
MCC	70	55			U	+	0	-	-	0
MCC	71	56			U	+	0	0	-	0
MCC	72	57	1		U	+	0	-	-	0
MCC	73		2		U	+	0	0	-	0
MCC	75	58			U	+	-	-	-	0
MCC	77	59	1	1	P	+	-	-	-	0
MCC	78		1	2	P	0	0	-	-	+
MCC	79		2	1	R	+	-	-	-	0
MCC	81	60			P	+	0	0	-	0
MCC	84	61			U	+	-	-	-	0
MCC	85	62			P	0	0	0	0	0
MCC	86	63			P	+	0	0	-	0
MCC	87	64			P	+	-	-	-	0
MCC	88	65			P	0	0	0	-	0
MCC	89	66			P	+	-	-	-	0
MCC	90	67			P	0	0	0	0	0
MCC	91	68			P	+	+	0	0	0
MCC	92	69			P	+	0	-	-	+
MCC	93	70			P	+	0	-	-	0
MCC	94	71			P	+	0	0	0	0
MCC	95	72			P	+	0	+	0	0
MCC	96	73	1	1	P	+	0	+	0	0
MCC	97		1	2	P	+	0	+	0	0
MCC	98	74			P	+	0	+	0	0
MCC	99	75			P	+	0	0	0	-
MCC	100	76			P	+	+	0	0	0
MCC	101	77			U	+	0	+	0	0
MCC	102	78			U	+	0	+	0	0
MCC	103	79			P	+	0	-	-	-
Contr	NH1					0	0	+	0	0
Contr	NH5					0	-	-	-	0

Table S2. *Cont.*

No		Patient	Tumor	Sample	Origin	MCPyV	RASSF2	RASSF5A	RASSF5C	RASSF10
Contr	NH6					0	0	0	0	0
Contr	NH7					0	-	0	-	0
Contr	NH8					0	0	+	0	0
Contr	NH9					+	0	+	0	0
Contr	NH10					0	0	0	0	0
Contr	NH12					0	-	-	-	0
Contr	NH13					+	-	-	0	-
Contr	NH15					+	-	-	0	-
Contr	NH16					0	0	-	-	0
Contr	NH18					+	-	-	0	-
Contr	NH19					0	-	-	-	0
Contr	NH21					0	0	-	-	-
Contr	NH22					+	-	0	0	-
Contr	NH23					+	-	-	0	-
Contr	NH24					0	0	-	-	0
Contr	NH27					+	-	-	0	0
Contr	NH29					0	0	-	-	0
Contr	NH30					0	-	-	-	0

MCC = Merkel cell carcinoma; Contr = normal tissue; P = primary carcinoma; R = recurrence; U = uncertain; + = positive; 0 = negative; - = not analyzed.
